# Reduced metagenome sequencing for strain-resolution taxonomic profiles

**DOI:** 10.1186/s40168-021-01019-8

**Published:** 2021-03-29

**Authors:** Lars Snipen, Inga-Leena Angell, Torbjørn Rognes, Knut Rudi

**Affiliations:** 1 Department of Chemistry, Biotechnology and Food Sciences, Norwegian University of Life Sciences, P.O. Box 5003, NO-1432 Ås, Norway; 2 Department of Informatics, University of Oslo, P.O. Box 1080, Blindern, NO-0316 Oslo, Norway

**Keywords:** Metagenome, Strains, ddRADseq

## Abstract

**Background:**

Studies of shifts in microbial community composition has many applications. For studies at species or subspecies levels, the 16S amplicon sequencing lacks resolution and is often replaced by full shotgun sequencing. Due to higher costs, this restricts the number of samples sequenced. As an alternative to a full shotgun sequencing we have investigated the use of Reduced Metagenome Sequencing (RMS) to estimate the composition of a microbial community. This involves the use of double-digested restriction-associated DNA sequencing, which means only a smaller fraction of the genomes are sequenced. The read sets obtained by this approach have properties different from both amplicon and shotgun data, and analysis pipelines for both can either not be used at all or not explore the full potential of RMS data.

**Results:**

We suggest a procedure for analyzing such data, based on fragment clustering and the use of a constrained ordinary least square de-convolution for estimating the relative abundance of all community members. Mock community datasets show the potential to clearly separate strains even when the 16S is 100% identical, and genome-wide differences is < 0.02, indicating RMS has a very high resolution. From a simulation study, we compare RMS to shotgun sequencing and show that we get improved abundance estimates when the community has many very closely related genomes. From a real dataset of infant guts, we show that RMS is capable of detecting a strain diversity gradient for *Escherichia coli* across time.

**Conclusion:**

We find that RMS is a good alternative to either metabarcoding or shotgun sequencing when it comes to resolving microbial communities at the strain level. Like shotgun metagenomics, it requires a good database of reference genomes and is well suited for studies of the human gut or other communities where many reference genomes exist. A data analysis pipeline is offered, as an R package at https://github.com/larssnip/microRMS.

Video abstract

**Supplementary Information:**

The online version contains supplementary material available at 10.1186/s40168-021-01019-8.

## Background

The study of microbial communities relies on the sequencing of microbial DNA, and current practice can be divided into two main approaches: metabarcoding, also known as amplicon or targeted sequencing, and shotgun sequencing of random fragments from the entire genome [1]. The amplicon approach is primarily used for revealing the taxonomic composition, but may also be used to study the distribution of targeted functional genes [2]. Shotgun sequencing provides a potentially more detailed information about the community genomes, the microbiome, and is typically used for studies that dig beyond the composition and into the genomic function. Shotgun microbiome sequencing requires significantly more efforts in sequencing, data processing, and analysis compared with metabarcoding.

In most microbiome studies, the composition is of interest, and in some cases, it is all we require. Shifts in composition may be used as indicators in various ways (e.g., microbiota profiles in forensics [3], or in the surveillance of environments) [4]. For communities like the human gut, extensive studies of the composition has given us the big picture, but recent investigations indicate that differences at the strain level may be crucial for phenotypic differences [5, 6]. Common to these problems is the need for high-resolution taxonomic profiles that can be collected with moderate efforts and in a reproducible way. Such studies often require many samples in order to capture the biological variation, and since sequencing and computational resources are always limited, the simpler amplicon approach is often preferred to a deep shotgun sequencing in order to get enough samples covered. However, the standard approach using the 16S rRNA gene marker has a limited resolution. If separation at the species or strain level is required, the 16S marker is in general too conserved, and a full shotgun sequencing seems necessary.

As an alternative to do a full shotgun metagenomic sequencing, the use of restriction enzymes to reduce the genomic sequence space has also been employed to investigate microbial communities [7–9]. The double-digested restriction-associated DNA sequencing (ddRADseq) idea [10–12] has been along for some time, but its use for metagenome studies is quite new. The main advantage for this approach has been to reduce the sequencing efforts, and thereby costs per sample. In short, this means cutting DNA into fragments using two different restriction enzymes, followed by a PCR amplification and sequencing of the resulting amplicons. This procedure falls between the full shotgun sequencing and the classical use of a specified marker gene (16S). It has some resemblance to shotgun sequencing, since from each genome we sequence many different, in some sense random, fragments that vary in size and number between genomes. However, this also resembles metabarcoding since multiple copies of a certain genome will produce the exact same fragments, and the reads are from these fragments each time. Thus, the approach has been termed Reduced Metagenome Sequencing (RMS). The wet-lab protocols for ddRADseq are well established [8].

There are some difficulties that arise with the RMS approach. When target sequencing a pre-defined marker gene like the 16S, we may cluster reads into operational taxonomic units (OTUs) or sequence variants, where each cluster represents some taxon. RMS reads may also be clustered, but each taxon gives rise to a variable number of distinct fragments, and it is difficult to infer the taxonomic composition from such read clusters without mapping to some references. Also, due to the variable lengths and compositions of the fragments, the PCR-amplification efficiency must be expected to vary and create biases. For purely predictive purposes, such reference-free approaches may still be useful, as suggested by [9].

In this paper, reads are in some way mapped to a database of reference genomes, often referred to as closed-reference analysis. Our focus is on the estimation of high-resolution abundances (i.e. species or strain-level profiles), which is not attainable by conventional 16S sequencing. It is possible to use computational tools designed for shotgun data directly in the RMS setting. This may produce helpful results, but do not utilize all the information we have in this case. We propose an alternative analysis approach, using fragment clustering and a constrained least squares estimation. Based on mock community data and simulations, we demonstrate some important aspects of RMS data, and show the potential for RMS to improve composition estimates at the strain level. We also include an example of using RMS to estimate strain diversity for *Escherichia coli* in the infant gut microbiome. The analysis tools, along with some tutorial material, is freely available as an R package at the GitHub site https://github.com/larssnip/microRMS.

## Results

**Fig. 1:**
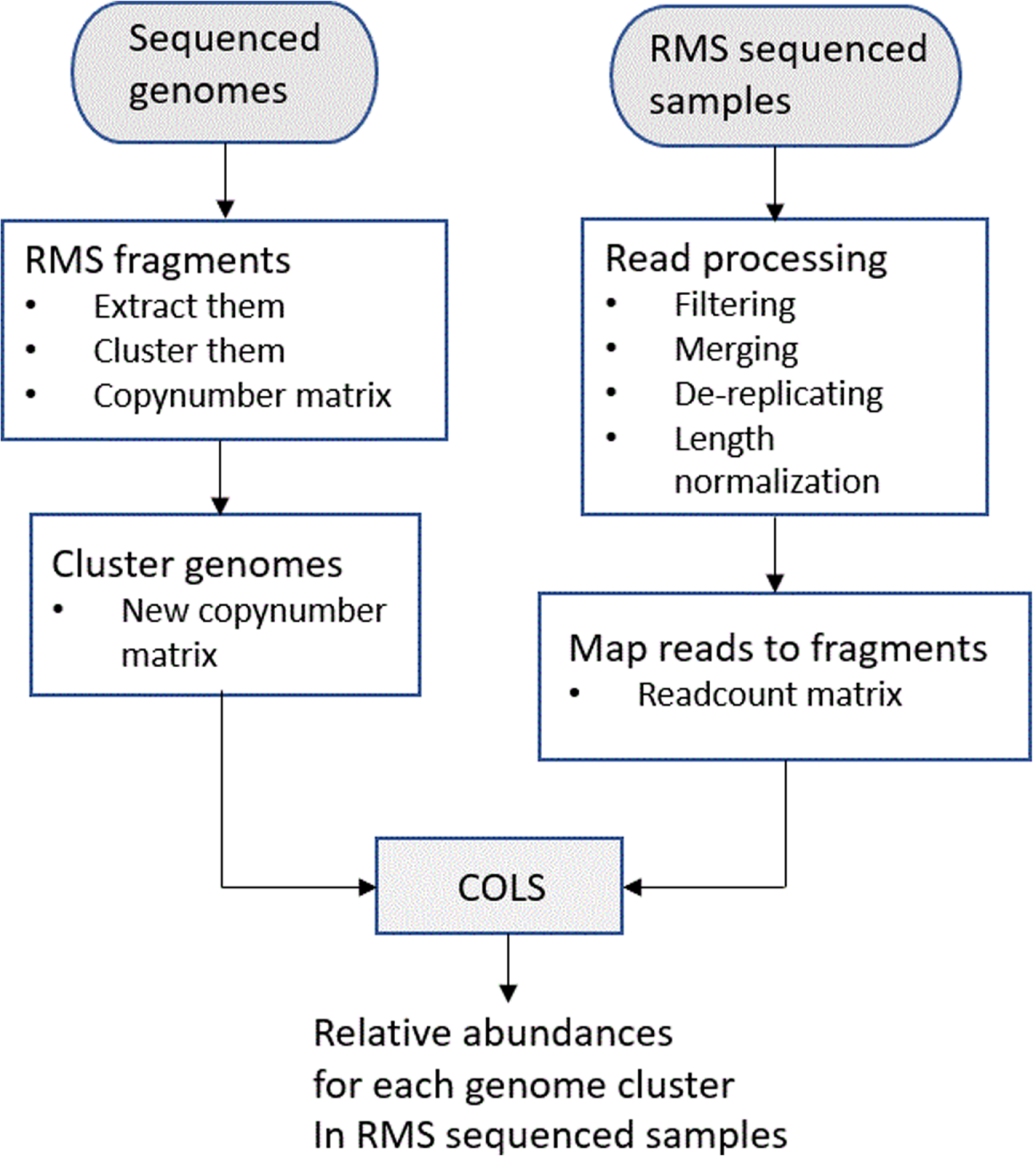
An illustration of the suggested RMS profiling procedure. The left branch is executed once for a collection of reference genomes, except that the clustering of genomes may be done at various resolutions depending on later use. The right branch is done for each set of samples. The supplied R package has a tutorial illustrating all steps

We have explored the Reduced Metagenome Sequencing approach for studying the composition of microbial communities, with a focus on high-resolution profiles. The RMS idea is to cut genomes into fragments using restriction enzymes, then amplify and sequence the resulting fragments. In this study, we have focused on the restriction enzymes EcoRI and MseI. The data processing pipeline illustrated in Fig. 1 will apply to any choice of enzymes, but some of the choices made along the way may change.

**Fig. 2:**
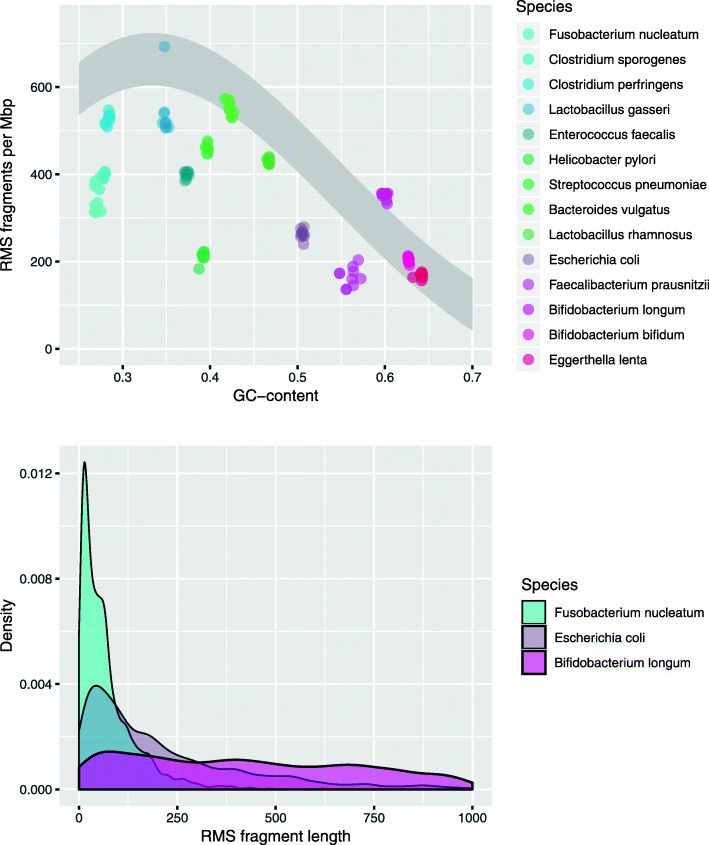
The distribution of RMS fragment number and lengths in some selected genomes, using the restriction cut sites GAATTC (EcoRI) and TTAA (MseI). There are 10 genomes for each of the species. In the upper panel, each dot corresponds to a genome, and the grey sector indicates where the dots should be if it was purely random DNA. In the lower panel, each density is based on data from 10 genomes

In Fig. 2, we see how the RMS fragment number and lengths distribute for a selection of species typically found in the human gut. This will clearly change if other restriction enzymes are used. For each species, we randomly selected 10 sequenced strains, and all results are based on retrieving the RMS fragments *in silico* from the genomes, using the cutting motifs GAATTC (EcoRI) and TTAA (MseI). In the upper panel, we notice that the number of RMS fragments per mega base pair varies a lot between species, but less within each species. The number of RMS fragments is typically limited by the occurrence of the longer cutting motif, in this case GAATTC. Since this is a guanine-cytosine (GC)-poor motif, there is an effect of GC-content, and the grey sector indicates where the numbers should have been had it been random DNA. In the lower panel, the densities show how fragment lengths distribute. Here, we selected three species only, having low (*Fusobacterium nucleatum*), medium (*E. coli*), and high (*B. longum*) GC-content. The fragment lengths are typically governed by the occurrence of the shorter motif, in this case TTAA, and again there is a huge effect of GC-content. The GC-poor *F. nucleatum* has very short fragments since the short motif occurs more frequently than in the more GC-rich species. There are fragments longer than 1000 bases, but these typically produce low signals after PCR amplification and are not shown here. For any choice of restriction enzymes, similar investigations should be made to see how many and how long fragments one should expect from the species most likely to be found in the targeted samples.

**Fig. 3:**
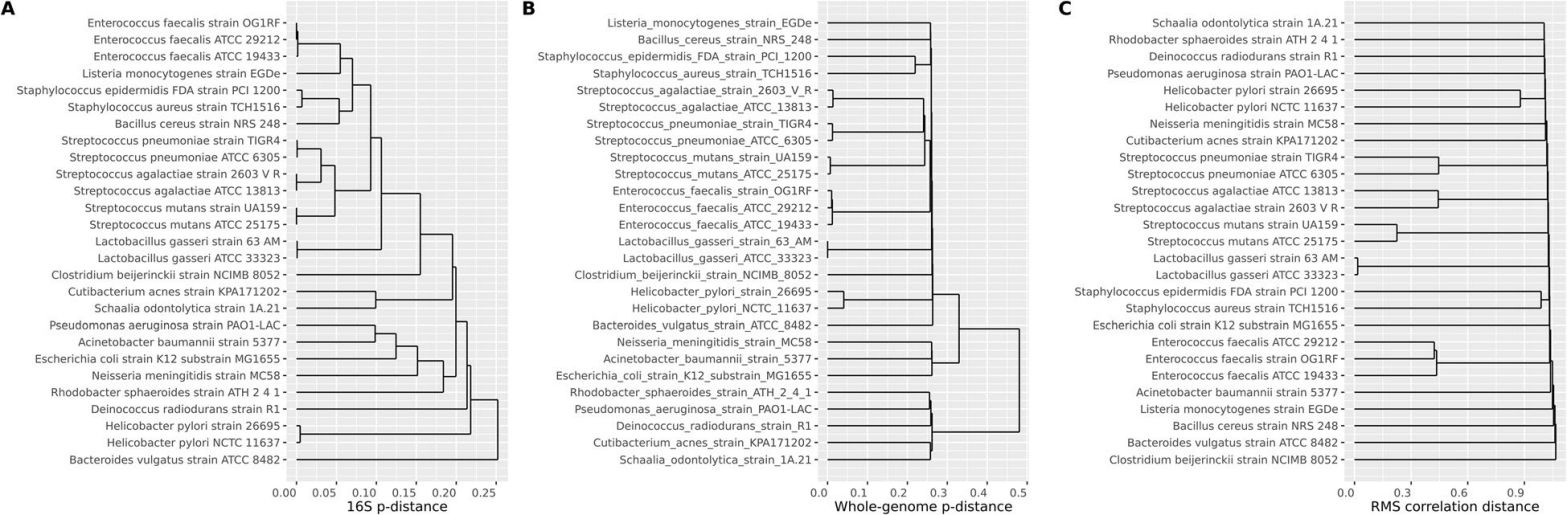
The dendrograms display hierarchical clustering of the 27 genomes in the mock study. In panel A, the distances are p-distances computed from a multiple alignment of the 16S sequences from each genome. In panel B, the p-distances are based on whole-genome comparisons, and in panel C, we used correlation distances based on RMS fragment copy numbers

Figure 3 illustrates the potential for high resolution using the RMS method. Here we have considered the 27 genomes used in our mock community below. In panel A, we used the 16S sequence from each genome. These were aligned (MUSCLE, [13]) and the p-distance (1 minus identity, i.e., distance 0.01 corresponds to 99% identity) between them computed using the ape-package in R [14]. We noticed that strains within the same species are identical or close to identical, and even the two *Staphylococcus* species are difficult to separate, with p-distance < 0.01. OTUs based on 16S data are usually clustered at distance 0.03. In panel B, we computed the p-distances based on whole genomes, using the fastANI software [15]. This separates the *Staphylococci* better than 16S, but strains within the same species are again quite similar. The two strains of *Helicobacter pylori* have p-distance 0.04 between them, indicating 96% of their genomes are identical. In panel C is the correlation distance between genomes based on RMS fragment copy numbers. From the genomes, we get the copy number matrix ***X***, with one row for each fragment cluster and one column for each genome. The two *Lactobacillus gasseri* strains have a small correlation distance, making them as good as impossible to separate with RMS. But, the other species with multiple strains are far better off, and the correlation distance of 0.22 between the two *S. mutans* strains should be large enough for discrimination between them.

**Fig. 4:**
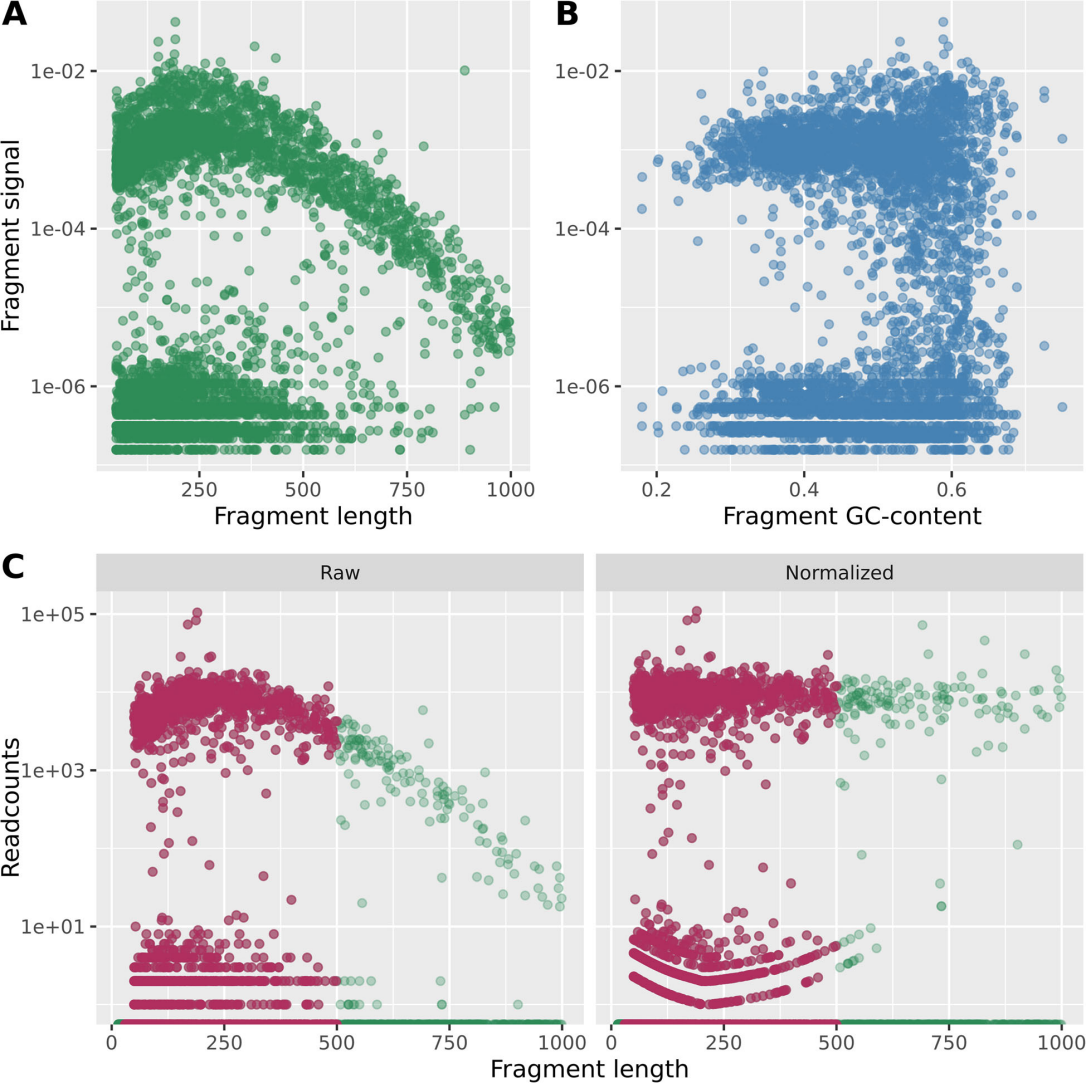
In panel A, fragment signal (relative read counts) is plotted against fragment length and in panel B against fragment GC-content. Each dot corresponds to a fragment cluster, and data from the four single-genome samples are displayed together. In panel C is shown the effect of the simple length normalization on a single sample. Raw read counts are normalized as described in the text. The red-brown color highlights the fragments within the length interval 30–500 bases. Note the log-transformed *y*-axes in all panels

Previous use of ddRADseq have reported biases in the signals, most likely introduced in the PCR amplification of such fragments [16]. To investigate this, we used mock data from [8], where some samples included a single genome only. In Fig. 4, we have plotted the data from four such samples. From panel A, we clearly see how fragment length affects the relative read count signals by the banana-shaped cloud of strong signals. The cloud of very low signals (note log-transformed *y*-axis) is due to noise. In panel B, there seems to be no bias due to GC-content. Based on repeated observations of similar patterns, we propose a length normalization of the RMS signals. In panel C, we show the effect of this procedure described in the Methods section. We also suggest, based on panels A and C, that only fragments within the length interval 30–500 bases should be used in the downstream analyses, highlighted in panel C of Fig. 4. In this interval, the length bias is small, and the normalization procedure will not affect the data very much, which is always a good thing. From Fig. 2, we also saw that most fragments are in this length interval when using the current restriction enzymes. Clearly, these limits must be reconsidered if other enzymes are used.

**Fig. 5:**
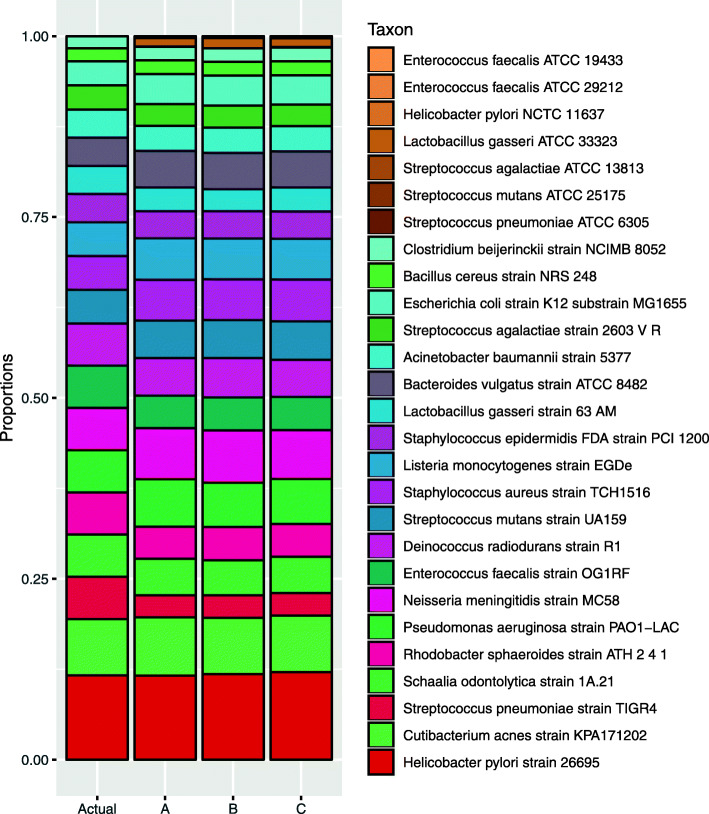
Actual and estimated composition of the mock with 20 genomes and no extra genomes spiked-in. The extra strains, absent from the samples, are indicated by the tan colors. The left bar is the actual composition, and the three additional bars (A, B, and C) show estimates from the three replicates of this sample

**Fig. 6:**
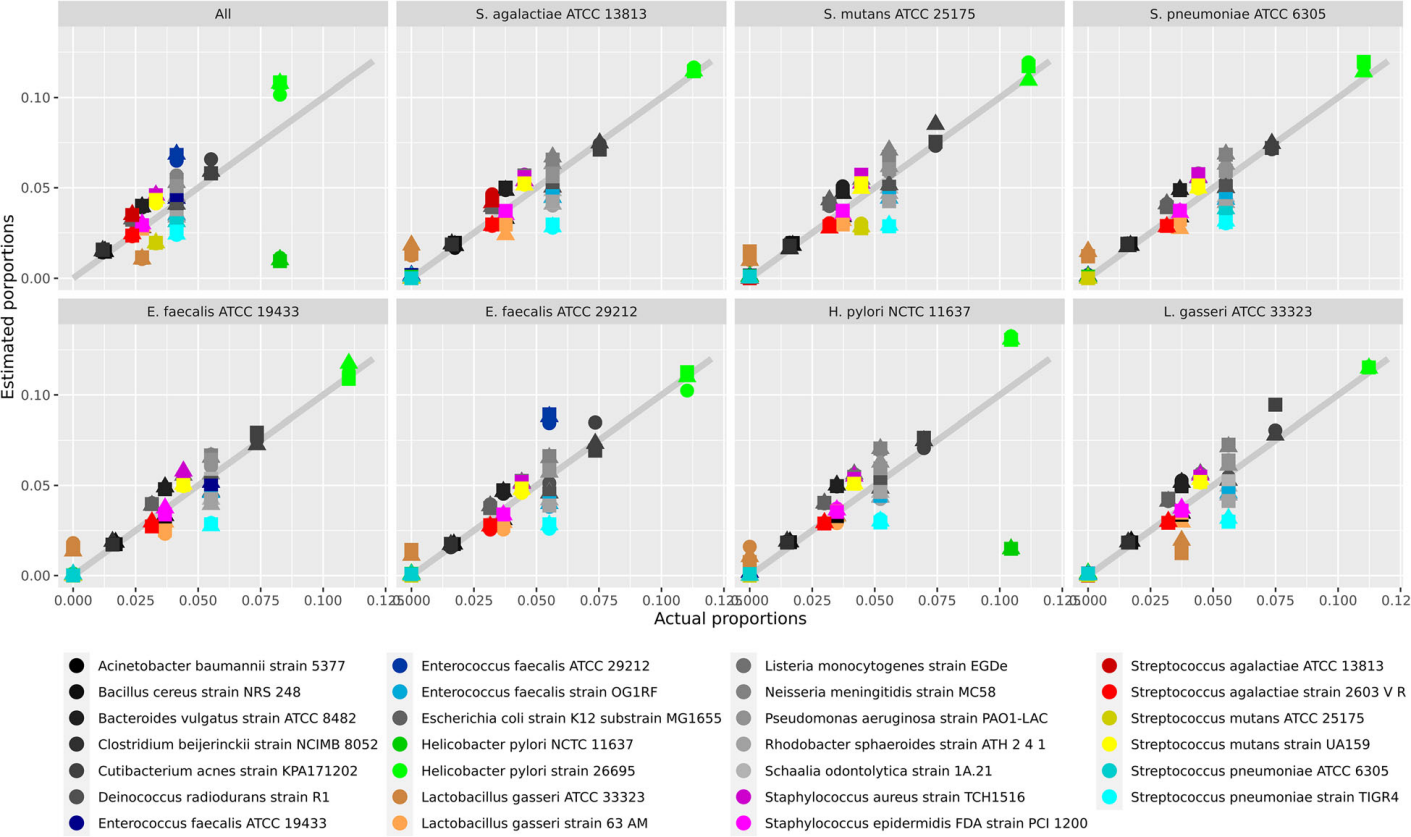
Each panel show actual versus estimated proportions for all 27 genomes as markers. Each marker color corresponds to a genome, and the three marker types are the three replicates. Species with a single genome is colored in shades of grey, while those where two or three genomes are similar have more distinct coloring. There are eight different mocks, all consisting of the 20 genomes in the original mock, but with various additional genomes spiked-in. The header above each panel indicates which genome has been spiked-in. The gray line in the background is where marker should be if the estimates were perfect. Note that in the lower left corner (those who are absent and predicted to be absent), many markers of different colors overlap each other.

Next, we used the RMS approach on the mock community data. In Fig. 5, we show a classical stacked bar plot displaying the estimated composition of the 20-genome mock. The estimates are based on the constrained ordinary least squares (COLS, see Methods for details) procedure, with 0.1 trimming, described in the Methods section. The database contains the genomes from all the 27 genomes, but only the original 20 genomes were included in this sample. Proportions are well estimated, and of the seven extra genomes absent in the samples, six of them are correctly estimated to have no contributions. The only false-positive (weak) signal is from *L. gasseri* ATCC 33323, as expected from the results in Fig. 3. In Fig. 6, we display the actual versus the predicted relative abundances as scatterplots for each of the other mock combinations. Here the extra strains were spiked-in, one by one, and in one sample, all seven were added. From this, we note that proportions are in most cases well estimated, where strains who are absent are estimated with zero proportions, and when strains are spiked-in they are estimated with fairly accurate proportions. There are two exceptions. As seen also in Fig. 5, the *L. gasseri* ATCC 33323 strain is estimated as (weakly) present also in those cases it is absent, and the spiking-in of *H. pylori* NCTC 11637 seems to have failed in some way, coming out with much too low abundance in the two cases where it is present.

**Fig. 7:**
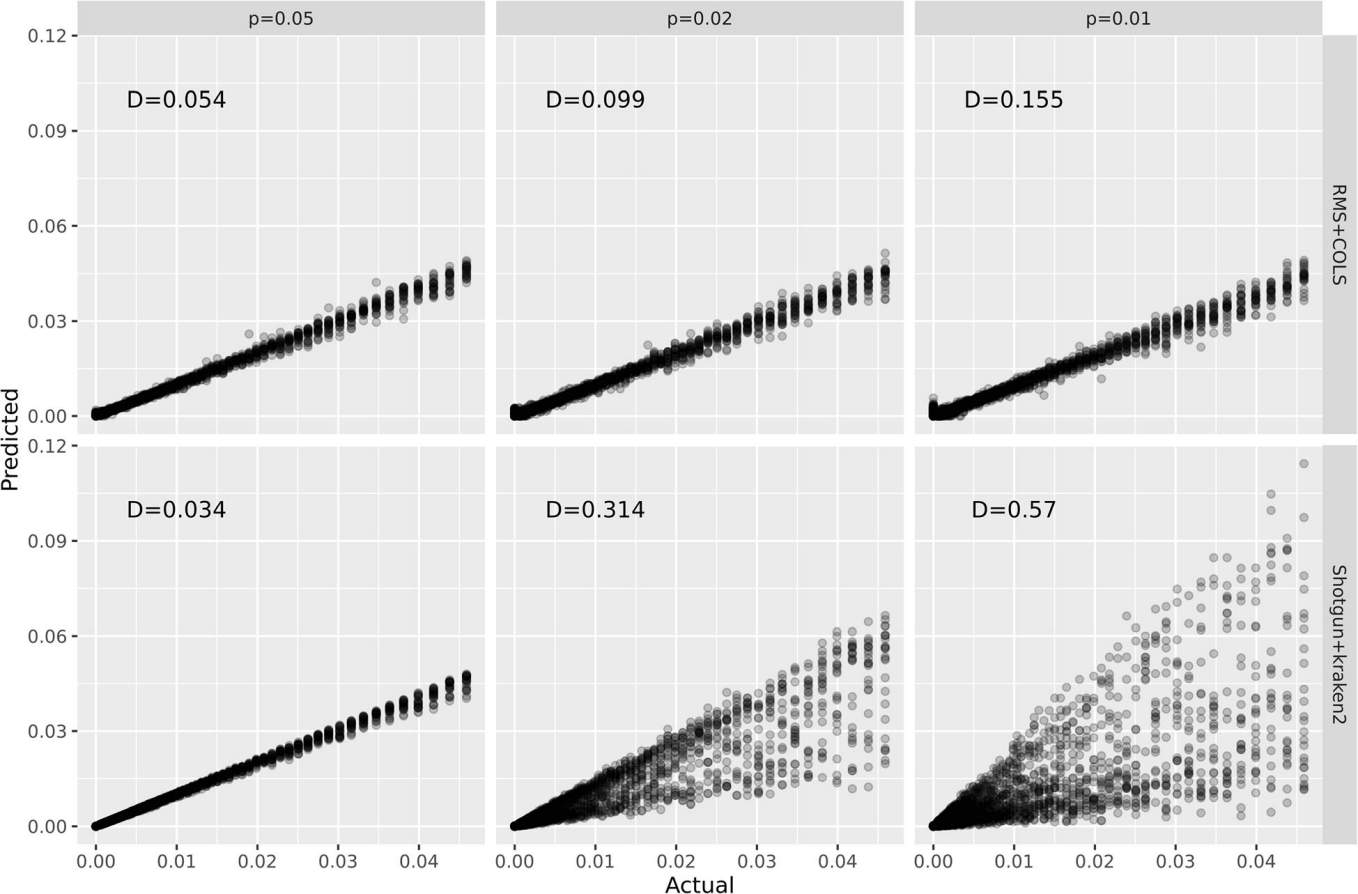
The scatter plots show actual versus predicted relative abundances for the simulated data. Each dot is a relative abundance of a genome, and each panel contain results from 25 samples. The upper panels are RMS data estimated by COLS, and the lower panels shotgun data estimated by Kraken2. The resolution of the communities increases from left to right, as indicated by the upper panel headers. The average Manhattan distance *D* is displayed within each panel

We also tested the RMS approach against a standard shotgun sequencing procedure using simulated data. We focused on human gut-like communities of three different resolutions, where community members have a minimum whole-genome p-distance of 0.05, 0.02, or 0.01. This resulted in 291, 601, and 1086 community genomes, respectively. In all cases, the samples contained reads from 100 randomly sampled present genomes, but the databases (RMS and Kraken2) contained all community genomes, both those present and absent. Both from RMS and shotgun data, we re-estimated the relative abundance of every single genome in the database, using the COLS method for RMS data and Kraken2 [17] with a custom database for the shotgun data. To evaluate the results, we computed the Manhattan distance (or L_1_ norm) between actual and predicted relative abundances, as suggested in [18]. Thus, a Manhattan distance of *D* = 0 means we estimate all relative abundances perfectly. In Fig. 7, the actual versus the predicted abundances are plotted as scatterplots. We observe, as expected, that predictions are poorer for lower p-distances (i.e., it becomes more difficult to distinguish genomes as they become more similar). However, the difference between RMS (upper panels) and shotgun (lower panels) data is striking. With the RMS approach, we can estimate the abundance of each genome quite well, while for shotgun data, the variance becomes huge for the highest resolutions, with predicted abundances up to three times larger or smaller than the actual abundances.

**Fig. 8:**
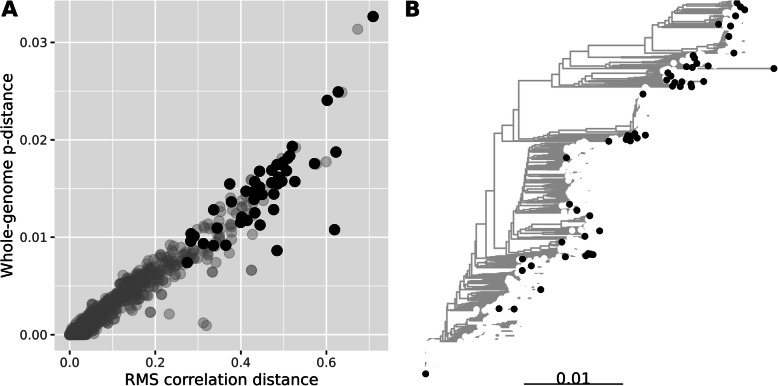
In panel A, each dot indicates distance to the nearest neighbor from an *E. coli* genome, measured either as RMS correlation distance (*x*-axis) or whole-genome p-distance (*y*-axis). The grey dots are the results for all 1066 genomes, and the black dots are for the 54 genomes left after clustering with maximum condition value 100. In panel B, the neighbor-joining tree is based on the p-distances between all strains, and the black tips indicate the cluster centroid genomes

Finally, we include some real data to illustrate our use of RMS. First, we use it for quantifying strain-diversity of *E. coli* in the infant gut. At the time of writing, there are 1066 complete *E. coli* genomes in the RefSeq database (https://ftp.ncbi.nlm.nih.gov/genomes/refseq/). This is an example of a genome collection where we find some very similar genomes. We computed the whole-genome p-distance between all pairs of genomes, using the MASH software [19], as well as the RMS correlation distances described in the Methods section. In Fig. 8, the left of panel A scatterplot indicates how these distances relate to each other. We only plot the distance to the nearest neighbor for each genome, and the grey dots are for all 1066 genomes. Note that some of distances are zero (both distance measures), indicating RefSeq contains multiple copies of identical genomes. The scatterplot also relates the correlation distance to the more familiar p-distance, and we observe that a correlation distance around 0.30 here corresponds to a p-distance of roughly 0.01, i.e., genomes of 99% identity.

Using all 1066 genomes turns out impossible, because the copy number matrix produces an infinite condition value. This is due to the identical genomes, which is (theoretically) impossible to separate. Hence, we employed the genome clustering described in the Methods section. Setting the maximum tolerated condition value at 100 produced 54 genome clusters, i.e., the *E. coli* population is divided into 54 subgroups with this resolution. The black dots in Fig. 7 are the nearest neighbor distances for these 54 genomes. In the right of panel B we indicate where these are located in a neighbor-joining tree based on the p-distances between all strains.

**Fig. 9:**
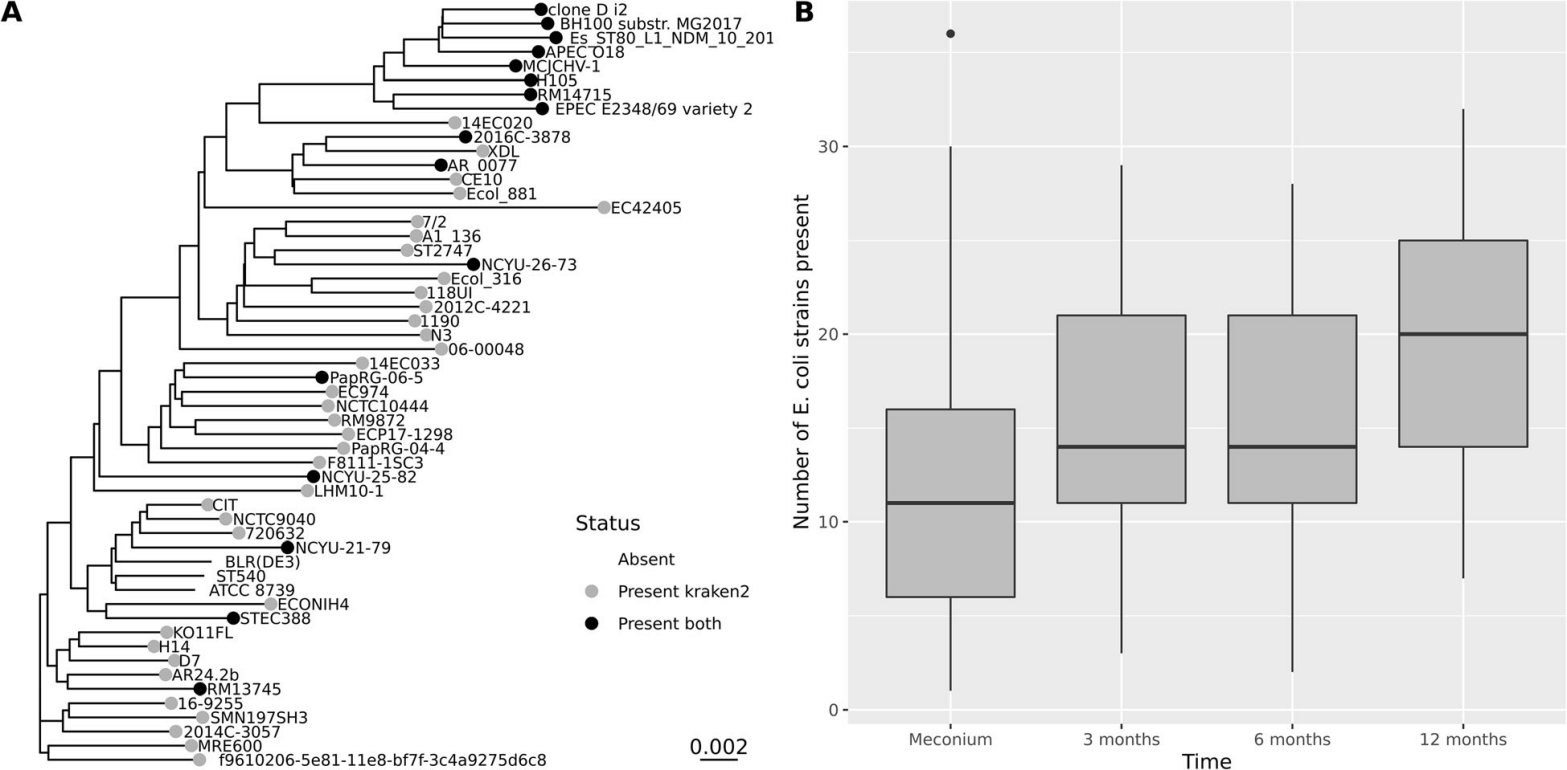
The left of panel A shows a tree for the 54 *E. coli* strains, based on the whole-genome p-distances, with a scale marker in the lower right corner. The leaf nodes are marked by how they were classified as present in a single sample. No leaf marker means classified as absent by both shotgun + Kraken2 and RMS + COLS. Grey markers indicate classified as present by shotgun + Kraken2 only. Black dots indicate classified as present by both methods. No genomes were classified as present by RMS + COLS only. In the right of panel B, the boxplot shows the number of genomes, out of the 54, estimated to be present by RMS + COLS over time in all infant gut samples (Meconium is newborn feces). There are 94 samples behind each box

Six of the samples from the infant guts were also subject to a conventional shotgun sequencing. We first made a comparison between shotgun and RMS, using Kraken2 and a custom database for assigning reads to the exact same 54 *E. coli* genomes that we used for the RMS analysis. Since we do not know the true composition of these samples, we only considered which strains were estimated to be present or absent in a sample. In the left of panel A of Fig. 9, we show a tree of the 54 strains, based on whole-genome p-distances, where we have colored the leaf nodes by how they were classified by either shotgun or RMS data in one of the samples. We can see that from the shotgun data, and the Kraken2 assignments, 51 of the 54 strains were assigned reads, and thereby being present (grey or black), while the RMS results only estimate 16 strains as present (black only), of which half is from the same clade at the top of the tree. The other five samples show a similar trend: The shotgun approach will assign reads to a majority of strains, while the RMS approach is more specific, stating fewer strains are present.

In the right of panel B of Fig. 9, the boxplot shows the number of strains found present by RMS in all 94 infants at 4 different times after birth. The variance is large, partly due to biological variation. Still, the trend of a growing diversity by age is clear, and a simple ANOVA analysis confirmed a highly significant increase in diversity from birth (Meconium) to all later times, especially to 12 months.

As a second illustration of the use of RMS and our deconvolution method, we re-analyzed the data from [7]. In their paper, they sequenced 3 human gut samples with both a conventional shotgun approach as well as an RMS approach. They used other restriction enzymes than we did in our analyses above, the NlaIII with cutting motif CATG and HpyCH4IV with ACGT. In [7], all data were profiled by MetaPhlAn2 [20], i.e., both shotgun and RMS data were treated the same way. In our reanalysis we used Kraken2 instead of MetaPhlAn2, and in addition, we also used our RMS-specific method on the RMS data. As a genome database, both for Kraken2 and our own method, we used the MGnify collection of human gut genomes [21]. This consists of 4644 genomes isolated from human gut samples and clustered at 95% identity, i.e., each genome represents a cluster of genomes with more than 95% identity.

**Fig. 10:**
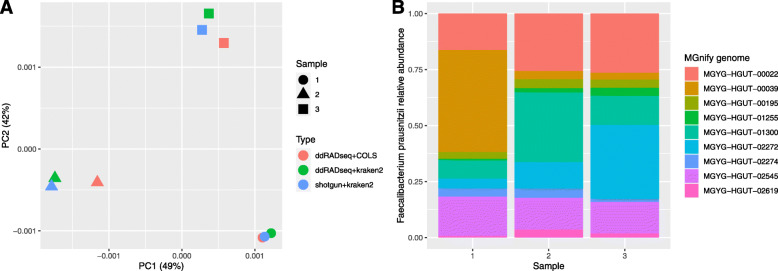
Reanalysis of the data from [7]. In the left of panel A, we compare the profiles obtained by using Kraken2 on the conventional shotgun (shotgun + kraken2) data and the RMS data (ddRADseq + kraken2). In the original paper, the same comparison was made using MetaPhlAn instead of Kraken2. In addition, we also used our approach described in this paper on the RMS data (ddRADseq + COLS). The marker-type indicates the samples, and the coloring the methods. In the right of panel B, we focus only on a strain resolution of the species *F. prausnitzii*, being the most dominant species in these samples. The nine strains listed are from the MGnify database (https://www.ebi.ac.uk/metagenomics/)

In the left of panel A of Fig. 10, we show a principal component analysis (PCA) plot of the three samples based on shotgun sequencing and Kraken2 profiling (shotgun + kraken), RMS sequencing and Kraken2 (ddRADseq + kraken2), and RMS sequencing profiled by our COLS method (ddRADseq + COLS). Only the 100 overall most abundant taxa were included to make the figure, and their relative abundances were transformed by the centered log-ratio transform [22] prior to the PCA computations and plotting.

In [7], the most abundant species identified by MetaPhlAn2 in these samples was *Faecalibacterium prausnitzii*. This is a common human gut species, but is also known to consist of multiple phylotypes, where different phylotypes have been reported to be associated with differing disease developments [23, 24]. Thus, it is of some interest to separate between variants of this species. In the MGnify collection, we find 9 different genome clusters named *F. prausnitzii*. We therefore used our deconvolution approach to separate between them, as plotted in the right of panel B of Fig. 10.

## Discussion

The upper panel of Fig. 2 shows that, using the restriction enzymes of this study, the RMS fragment density varies by GC-content. Most genomes also have fewer fragments than expected in random DNA, indicating a negative selection of the cut sites. However, multiplying by genome size, we find that most genomes have in the range of hundred to thousand fragments. The lower panel of Fig. 1 shows most fragments are rather short. This is a good thing, since longer fragments amplify poorly, and we found that by only focusing on fragments in the length interval 30–500 bases, we obtain strong signals without too much PCR bias. These results apply to our chosen restriction enzymes and should always be investigated for any alternative choices of enzymes.

From Fig. 3, we clearly see the potential for RMS to resolve strains at a level which is impossible with 16S, and even difficult with shotgun sequencing. In panel A, we used full-length 16S sequences, but still the separation is very poor between closely related genomes. The distances in panel B reflects how similar the genomes are in overall nucleotide identity. As expected, strains within a species have p-distance of less than 0.05, i.e., more than 95% identical. Panel C demonstrates that even closely related strains have rather large correlation distance, indicating a good number of unique RMS fragments. This may seem strange, how can genomes be so similar in p-distance, but still have different RMS fragments? Mutations in restriction cut sites as well as rearrangements of genomic regions will both create/destroy RMS fragments, but have little impact on the whole-genome distance. Only if two or more genomes share the vast majority of RMS fragments, we would see a small correlation distance, and a shallow branch in the dendrogram. From panel C in Fig. 2, we expect to be able to separate all genomes by RMS, except perhaps the two *L. gasseri* strains.

Since the RMS approach involves a PCR step, we must expect some biases. In Fig. 4 (panel A), we observe a distinct effect of fragment length on the relative signal strengths we get, based on single-genome samples from a previous study. However, the GC-content of the fragments does not seem to have any effect (panel B), unlike what was reported by [16]. The lower panels of Fig. 4 illustrate our proposed way of handling the length bias. First, only use fragments in the length interval 30–500 bases, highlighted in brown color in panel C. As we saw in Fig. 2, short fragments account for the vast majority anyway, and we have found that 80–90% of the reads will map to fragments in this interval. Next, we propose a simple normalization, illustrated in panel C. The cloud of strong signals is straightened. Note that due to the log-transformed *y*-axis it looks like weak signals (noise) are heavily distorted by the normalization, while their values actually change very little. If other restriction enzymes are used, the fragment lengths may be different, and the limits of 30–500 should be reconsidered. However, the length bias corrected by the normalization will probably be of the same type, since this is a PCR effect which is independent of the restriction enzymes.

The mock data results in Figs. 5 and 6 reveal that with the RMS approach and the COLS algorithm, we can estimate relative abundances fairly well. It should be noted that the actual abundances are probably not exact, as they rarely are in experimental data. The mock composition was designed by 16S copies, and the transformation to genome copies is not without uncertainty, since most of these species are known to have variable 16S copy numbers. Most important is that strains who are absent from a sample are also estimated to zero abundance, i.e., they show up in the lower left corner of the panels in Fig. 6. The exception is *L. gasseri* ATCC 33323. This is simply too similar to the other *L. gasseri* strain, as seen in Fig. 3 as well. Only five fragment clusters are unique to the ATCC 33323 strain, and with some noise signals on some of these, it appears to be present even when it is not.

In virtually all cases, the three replicates (marker types) of each sample show very similar results, indicating there is very little variance in the RMS procedure as such. Hence, any deviations between actual and estimated proportions are most likely due to some systematic effect. On closer inspection, we found that the RMS data display what we denote as a fragment bias. Fragments unique to a genome should in theory all produce similar read counts, with some variation due to the randomness of sequencing. This is not the case. Some fragments consistently produce strong signals and others weak. This is also remarkably stable across all samples where a particular genome is present. We accounted for this in our simulation study, adding a random scaling to all fragments, and supplementary Figure 2 shows the distribution of this fragment bias. So far, we have failed to reveal the cause of this effect. If we could understand and compensate for it, this would improve the precision and thereby the resolution of the method even further.

We used simulated data to compare the RMS approach to the use of shotgun sequencing in combination with the Kraken2 tool for re-estimating the relative abundances of each genome. Kraken2 is only one out of several tools for estimating metagenome composition, but we chose this because it has a good reputation, will always try to classify reads to the genomes of its database, but most importantly, the genomes in the database can be easily customized. To make the comparison fair, the database must be identical for both the RMS and shotgun approach. Using a generic database is bound to produce poorer results compared to one where the exact genomes under study are in the database. An alternative tool like MetaPhlAn2 [20] assigns reads no lower than the species level, but has the extension StrainPhlAn [25] for a strain level analysis. It is, however, difficult to compare StrainPhlAn output to the ones we get here, since we focus on relative abundances of *a priori* defined genomes, while StrainPhlAn identifies strains *a posteriori* by aligning reads to a set of marker genes and output a multiple sequence alignment. It seems to us these are two quite different approaches to a strain-level analysis.

In Fig. 7, we show some results of our simulation study. The left panels are from a community where no members have a p-distance below 0.05 to another member. This is roughly a community with one genome from each species. Here, both methods perform extremely well, and the shotgun + Kraken2 is the best, with Manhattan distance *D* = 0.034. However, as the communities (and databases) are filled up with more and more similar genomes, the picture changes (middle and right panels). The shotgun + Kraken2 results are getting dramatically poorer, with highly fluctuating estimates of relative abundances. The RMS + COLS approach is also poorer, but not nearly as bad. While the Kraken2 results seem to be fairly unbiased, but with a huge variance, the COLS results have a small variance, at the cost of some bias in giving weak abundance to some absent genomes, seen in the lower left corners of the 98 and 99% panels. Our explanation for these results is that with shotgun sequencing, most reads will match multiple genomes in the database, and Kraken2 will then assign to the lowest common ancestor, i.e., the species. Thus, species abundances become extremely precise, but too few reads are left at the strain level to get reliable estimates. In our COLS algorithm for RMS data, we also have many fragment clusters who are present in multiple genomes, but since we have the copy number matrix with this exact information, the constrained least square solution spreads the signal across all genomes instead of assigning it to their common ancestor. It should be mentioned that this idea has some resemblance to what was proposed by [26], using methods from RNAseq data as an alternative approach for analyzing shotgun data. The Kraken2 software also has an extension in the Bracken software [27], re-estimating low-rank abundances based on the higher-rank assignments, but is difficult to use below the species rank.

There is, as for any method, a limit to the resolution obtained by RMS. The example with 1066 *E. coli* genomes illustrates this. Many of these are more than 99% identical, some even 100%, as seen in Fig. 8. We plotted the correlation distance between all genomes against the p-distance for the same pairs, to illustrate how they are related. An RMS correlation distance of around 0.30 corresponds roughly to a p-distance of 0.01 (99% identity) in this case. We employ a genome clustering, where we only keep a selection of the genomes, ensuring a minimum difference between them. This means each cluster centroid represents a subgroup of highly similar strains. When using the COLS algorithm, the resolution is limited by the condition value of the fragment copy number matrix. A very large condition value indicates the estimated abundances will be unstable. Condition values of 10^2^, 10^3^, or even 10^4^ may be used to obtain a gradually higher resolutions, but at the cost of more uncertain results. Even with the lowest threshold at 10^2^, we get 54 subgroups in the analysis, and it is likely that in many cases such a resolution will suffice. As seen in Fig. 8, these strains are typically 98–99% identical (p-distance 0.02–0.01) and represent the full tree of all strains quite well.

A shotgun sequencing should in theory be able to separate anything below 100% identical, but in practice not. Reads are not without errors, and read coverage is often poor for low-abundance taxa. The results in Fig. 9 underlines this. The shotgun data indicate almost all genomes in the database are present in the sample. With the RMS approach, much fewer genomes are present. In one out of the six comparable samples, the methods came out with the exact same genomes as present and absent. In the others, the shotgun data always results in more detected strains. It is reasonable to suspect both methods are too sensitive, assigning too many subgroups as present, but RMS seems far better in this respect. The total fraction of *E. coli* is small in these samples (around 1%), but the absolute number of reads assigned to this species are in the same range for both methods (around 1000). It is in fact slightly larger for the RMS data; hence, the increased prevalence from shotgun data is not due to increased coverage. For shotgun data reads can originate from all locations on the genomes, making it notoriously difficult to map a read correctly when genomes are as similar as here, and given that reads may contain errors. RMS reads are assigned to the *a priori* known fragments and allow for some slack due to sequencing error. Also, if genomes A and B share 50% of their fragments, but only the genome A fragments have signal, the COLS algorithm will assign abundance 0.0 to genome B even if 50% of its fragments have signal. This is possible because we know these fragments are shared with genome A, and since the unique genome A fragments have signal while the unique genome B fragments have none, the shared fragment signals are all allocated to genome A, giving no abundance to genome B.

The boxplots in panel B of Fig. 9 is an example of how we use RMS to detect a change in strain diversity over time in the infant gut. The increasing diversity by age is as expected. This example also illustrates how patterns emerge because we were able to sequence many samples, rather than deep sequencing of a few, where the biological variation probably would obscure the results. Such a high-resolution analysis would not be possible by 16S analysis.

The reanalysis of the data from [7] is an example of using completely different restriction enzymes. The two four-base cutters result in far more fragment per genome than we saw in Fig. 2, but apart from this, the analysis we did was identical to what we have done above. The left panel of Fig. 10 shows that shotgun data and RMS data, assigned both by Kraken2 and our algorithm, result in the same big picture. The difference between methods is small compared to the difference between samples, which is the same conclusion reached by the authors of the original paper. In the right panel, however, we show that with our RMS-specific deconvolution, we may now also estimate abundances below the species level. The original results, using MetaPhlAn2, did not dig beyond the species level, but this seems to some degree possible with the approach we have suggested in this paper. The species *F. prausnitzii* is exactly a species where such analyses may be of some interest. We observe some difference in strain abundances here, but three samples are of course far too few to reach any conclusion along this road.

The RMS has been proposed as a low-cost alternative to a full shotgun sequencing [7], since we only sequence the amplified fragments accounting for a fraction of the entire genomes. This is true if you use a reference-free approach where you need to cluster the reads, and hence need to have sequenced the same region of a genome several times in order to say something about abundance. However, as long as reads are mapped to reference genomes, this difference in library complexity is less important. Instead, the potential gain in using RMS lies in precise estimates of strain resolution profiles. As for shotgun data, there is no theoretical lower sequencing depth that is required, the more reads the better. For the mock data results in Figs. 5 and 6, where strains separated nicely, each sample had between 1 and 2 million reads mapped to some fragments, resulting in mostly 10–100 reads per fragment. This we consider a very good coverage. As always, high coverage is needed for detecting low-abundance taxa, but is not in itself required for separating closely related strains. A bottleneck for RMS is the fragment bias previously mentioned. For some reason, fragments from the same genomes tend to get quite different read counts, in a reproducible way. If a genome has as very few fragments, the average read count for these is not as stable as with many fragments.

We believe our results indicate the RMS approach for metagenome profiling is something to explore further. We have focused a lot on one pair of restriction enzymes in this study, but other enzymes are used for similar studies [7, 9]. The choice of enzymes will affect the number and length of fragments, but apart from this, the data analysis procedure we propose here may be used, as we illustrate by the reanalysis of the data from [7]. In the supplied software (R package), there are options for using any pair of restriction enzymes. The RMS approach, like the shotgun metagenome approach, requires sequenced reference genomes to map against in order to produce taxonomic profiles. To obtain this at the strain level, we need good reference databases. The good news is that recent extensive efforts provide us with many new reference genomes, especially for the human gut [28–32]. We believe that with evolving sequencing technologies, the quality of metagenome-assembled genomes (MAGs) will improve drastically, and the road lies open for more strain-level profiling.

## Conclusion

We have demonstrated that the RMS approach can be used for profiling of microbial communities down to the strain level. Compared with the conventional 16S approach, we find that strains with identical 16S genes are clearly discriminated by RMS, and we can estimate abundances for such strains in the same sample. The reason for this is simply that even genomes with identical 16S sequences will in most cases differ in a fair number of RMS fragments, enough to obtain strain-specific signals for the COLS algorithm.

Compared with the shotgun metagenome approach, the RMS offers an advantage in only sequencing *a priori* known amplicons, and we may construct a copy number matrix revealing the relations between all reference genomes prior to any sequencing. From this information, and the suggested constrained ordinary least squares estimation algorithm, we can obtain strain-level abundance estimates at least as good as the popular metagenome tool Kraken2. A clustering of genomes into species subgroups is proposed, as a way of balancing high resolution against precision in estimated abundances.

Based on this, we conclude that the RMS approach is worth pursuing, as a tool for studies of composition in the human gut or other microbial communities of particular interest and where a comprehensive collection of reference genomes exists. An R-package with the data analysis methods suggested here, as well as tutorials, is available in GitHub at https://github.com/larssnip/microRMS.

## Methods

### Mock data

In order to test the RMS approach, and learn about how such data behave, a mock community study was conducted. As a basis, we used a mock community of 20 genomes obtained through BEI Resources, NIAID, NIH as part of the Human Microbiome Project (Genomic DNA from Microbial Mock Community B (Even, Low Concentration), v5.1L, for 16S rRNA Gene Sequencing, HM-782D, [33]) (see Table 1). This mock has been constructed to yield 100,000 16S copies from each included organism. We converted this into the number of genome copies by dividing 100,000 by the 16S copy number for each organism, as listed in the Ribosomal RNA Database [34]. In addition to this mock itself, we spiked-in 7 additional DSMZ strains (Leibniz Institute DSMZ-German Collection of Microorganisms and Cell Cultures, https://www.dsmz.de/). These strains were selected to be highly similar, and with identical 16S gene, to one of the existing strains in the mock, to see if we could separate signals from such closely related organisms. One strain was spiked-in at a time, producing 7 additional samples. The spiked-in genomes were also at 100,000 16S copies, controlled by a droplet digital PCR procedure. Finally, a sample with all 27 strains was also used. All these 9 mock mixtures where done in triplicates, resulting in 27 samples. All samples were subject to the wet-lab procedures described in [8] to obtain paired-end Illumina HiSeq reads. The restriction enzymes EcoRI and MseI were used throughout this study. All the strains involved in all samples have whole genome sequence data publicly available, and these were downloaded from the NCBI Genome database (https://www.ncbi.nlm.nih.gov/genome).

**Table 1:** For each genome is listed its size (megabasepairs), GC-content and the number of RMS fragments in the 30–500 length interval. Genomes with an asterisk (*) after its name were spiked-in, and not part of the original mock

Genome	Size	GC	RMS-fragments
*Acinetobacter baumannii* strain 5377	3.98	0.39	961
*Schaalia odontolytica* strain 1A.21	2.39	0.65	92
*Bacillus cereus* strain NRS 248	5.22	0.36	2025
*Bacteroides vulgatus* strain ATCC 8482	5.16	0.42	2047
*Clostridium beijerinckii* strain NCIMB 8052	6.00	0.30	2463
*Cutibacterium acnes* strain KPA171202	2.56	0.60	300
*Deinococcus radiodurans* strain R1	3.06	0.67	115
*Enterococcus faecalis* ATCC 19433*	2.87	0.38	902
*Enterococcus faecalis* ATCC 29212*	3.01	0.37	922
*Enterococcus faecalis* strain OG1RF	2.74	0.38	822
*Escherichia coli* strain K12 substrain MG1655	4.64	0.51	920
*Helicobacter pylori* NCTC 11637*	1.60	0.39	233
*Helicobacter pylori* strain 26695	1.67	0.39	255
*Lactobacillus gasseri* ATCC 33323*	1.82	0.35	662
*Lactobacillus gasseri* strain 63 AM	1.89	0.35	675
*Listeria monocytogenes* strain EGDe	2.94	0.38	1504
*Neisseria meningitidis* strain MC58	2.27	0.52	332
*Pseudomonas aeruginosa* strain PAO1-LAC	6.26	0.66	143
*Rhodobacter sphaeroides* strain ATH 2 4 1	4.13	0.69	92
*Staphylococcus aureus* strain TCH1516	2.88	0.33	861
*Staphylococcus epidermidis* FDA strain PCI 1200	2.50	0.32	854
*Streptococcus agalactiae* ATCC 13813*	2.11	0.35	661
*Streptococcus agalactiae* strain 2603 V R	2.16	0.36	692
*Streptococcus mutans* ATCC 25175*	1.99	0.37	671
*Streptococcus mutans* strain UA159	2.03	0.37	680
*Streptococcus pneumoniae* ATCC 6305*	2.02	0.40	709
*Streptococcus pneumoniae* strain TIGR4	2.16	0.40	771

### Infant gut data

As an illustration of a high-resolution analysis, we used a set of RMS data from the gut of infants. The microbiome was sampled from feces of 94 infants at meconium (newborn) and 3, 6, and 12 months age. We used a genome collection consisting of all complete RefSeq genomes of *E. coli* (1066 genomes) in order to look at strain diversity in these samples. Six of the samples were also sequenced by conventional shotgun sequencing, for comparison. All RMS samples were subject to the wet-lab procedures described in [8]. Both RMS and shotgun samples were sequenced by Illumina HiSeq, resulting in 150 bp paired-end reads.

### Fragment copy number matrix

There exists a number of computational tools for estimating the taxonomic composition of a community based on shotgun data, e.g., Kraken2, MetaPhlAn2, CLARK, Kaiju [17, 35–37]. Common to all is that reads are somehow mapped to some database of reference genomes. This is also required for RMS data. Given the reference genomes, and the cutting patterns of the restriction enzymes used (EcoRI and MseI), all RMS fragments were collected *in silico* from each genome. The RMS fragments are simply all subsequences starting with an EcoRI motif GAATTC (5’ of fragment), and ending by the first downstream MseI motif TTAA, containing none of these motifs inside. Genomes will, in general, have many such RMS fragments of highly variable lengths.

Fragments from closely related genomes may be identical or very similar. Also, some fragments may occur multiple times within a single genome. For this reason, we clustered the fragment sequences into fragment clusters, using the VSEARCH software [38] and some specified identity threshold, similar to OTU-clustering for 16S data. An identity threshold of 0.99 was used in this study, but other thresholds were tested without significant changes in results. Each fragment cluster was represented by its centroid sequence and the fragment cluster copy number was stored in a *copy number matrix*. This matrix {***X***} has one row for each fragment cluster and one column for each genome, and the integer in cell ***X****(i,j)* is the number of fragments from genome *j* that belongs to cluster *i*. If we have a large collection of genomes, this matrix becomes huge. However, most fragment clusters occur in only one or a few genomes, and most cells in the matrix are zero. Thus, the copy number matrix was stored as a sparse matrix data type, allowing most matrix operations but using comparatively little memory. This copy number matrix is an essential ingredient in the estimation of community abundances, as described below.

### Read processing

We used the software VSEARCH [38] for all processing of reads. All reads were subject to a quality filtering, keeping only reads with an expected error rate below 0.02. Read pairs were then merged. Since RMS fragments vary in length, some longer fragments produce non-overlapping reads. Thus, non-merged reads were included as single reads, where the R2 reads were reverse-complemented. To maintain the correct per-fragment read count, all merged reads were given a count of 2, while the single reads count as 1. All reads were then de-replicated to obtain fasta-files of unique reads for all samples. Proper use of the --sizein and --sizeout options in VSEARCH allows us to work with the smaller set of unique reads without losing any information about actual read abundances.

Next, the processed reads from each sample were mapped to the fragment cluster centroids, using VSEARCH and the identity threshold from the fragment clustering (0.99, see above). This produced a read count matrix ***Y***, with one row for each fragment cluster and one column for each sample.

### Length normalization

We realized the need for correcting the read count signals due to fragment-length PCR bias. First, let ***y***_*k*_ denote raw read counts from sample *k*, i.e., column *k* in ***Y***. Thus, ***y***_*k*_*(i)* is the raw read count for fragment cluster *i*, and *L*_*i*_ is the length of cluster centroid *i*. Setting aside all clusters with zero signal, the *c*_*i*_ *= log*_*10*_*(****y***_*k*_*(i))* is simply the log read count. Next, we fitted a locally weighted scatterplot smoother (loess) *S(c*_*i*_*|L*_*i*_*)* to these data, thus *S(c*_*i*_*|L*_*i*_*)* is a smooth curve describing how log read counts *c*_*i*_ vary by fragment length *L*_*i*_. Then, a correction factor for fragment cluster *i* is given as

$$ {f}_i={10}^{\left(S\max -S\left( ci| Li\right)\right)} $$

where *S*_max_ is the maximum value on the loess curve. The normalized read count for any fragment cluster is then

$$ {{\mathbf{y}}^{\ast}}_{\mathrm{k}\left(\mathrm{i}\right)}={\mathbf{y}}_{\mathrm{k}\left(\mathrm{i}\right)}\ {\mathrm{f}}_{\mathrm{i}} $$

This multiplicative adjustment means fragments with zero signal remain zero also after normalization. This normalization is done for each sample separately. If the database contains a huge number of fragment clusters (many genomes), only a random subsample of them may be used to fit the loess model in order to save time and memory.

### Constrained ordinary least squares (COLS)

If all fragment clusters were unique to a single genome, the abundance of each genome would naturally be estimated by averaging the read counts for their corresponding fragment clusters. However, many RMS fragment clusters may be found in several genomes, and more closely related genomes will share more fragment clusters.

Prior to sequencing, we constructed the copy number matrix from the *G* genomes in the database. This results in *C* fragment clusters; thus, the copy number matrix ***X*** has *C* rows and *G* columns. Let ***b*** *= (b*_*1*_*, b*_*2*_*,...,b*_*G*_*)* be the proportion of the various genomes in sample *k* (i.e., *b*_*j*_ *≥ 0* and ${\sum}_{j=1}^G{b}_j$ *=* 1). Then, it is reasonable to assume that

$$ \mathrm{E}\left({\mathbf{y}}_{\mathrm{k}}\right)=\mathrm{a}\ {\mathbf{X}}^{\mathrm{t}}\ \mathbf{b} $$

where ***y***_*k*_ are the (normalized) read counts for each of the *C* RMS fragment clusters given the data from sample *k*, ***X*** is the copy number matrix, and *a* is some positive scaling factor relevant for sample *k*. Thus, the expected signal for fragment cluster *i*, *E(****y***_*k*_*(i))*, is proportional to the linear combination of fragment cluster copy numbers and genome abundances.

Given this model, the scaled proportions can be estimated using the constrained ordinary least squares (COLS) approach: Find ***s*** *= a****b*** that minimizes

1 $$ f\left(\boldsymbol{s}\right)={\left({\boldsymbol{y}}_k-{\boldsymbol{X}}^t\boldsymbol{s}\right)}^t\ \left({\boldsymbol{y}}_k-{\boldsymbol{X}}^t\boldsymbol{s}\right)\kern2.25em \mathrm{where}\kern0.75em {s}_j\ge 0 $$

From the requirement that the ***b***'s must sum to 1.0, we get that $\sum \limits_j{s}_j=a$ and the estimated relative abundance of each genome is

$$ \hat{\boldsymbol{b}}=\frac{\hat{\boldsymbol{s}}}{\sum \limits_j{s}_j} $$

In the implementation of this de-convolution, we have added the possibility of a trimmed estimate. This means a two-stage estimation procedure: After the initial fitting of the model, as described above, the residuals ***y***_*k*_ *−* ***X***^*t*^$\hat{\boldsymbol{s}}$ for all fragment clusters are computed. Then, a user-selected fraction of the fragments with most extreme residuals are discarded, and the model is re-fitted on the trimmed fragment set. This makes the estimated abundances less sensitive to extreme signals from some fragments, but also reduces the size of the dataset.

### Correlation distance and genome clustering

The COLS algorithm also indirectly suggests the maximum resolution possible to de-convolve. If two genomes are very similar, they will share most RMS fragment clusters, and their respective columns in the copy number matrix ***X*** become similar. The *correlation distance* between two genomes is simply 1 minus the correlation between their respective columns in ***X***. Thus, a correlation distance of 0.0 means the two columns are identical, and the genomes share all fragment clusters. A correlation distance can be maximum 2.0, meaning all fragments present in one genome is absent in the other, and vice versa.

When solving eq. (1) we need to invert the matrix ***X***^*t*^***X***, and if two or more columns are too similar, this matrix inversion becomes highly unstable resulting in poor abundance estimates. This instability is often quantified by the condition value of ***X***^*t*^***X***. A perfect condition value of 1.0 means all columns in ***X*** are orthogonal (i.e., no shared fragments). As columns become more and more correlated, the condition value increases. By computing the condition value from ***X*,** we get an idea of how solvable this is, prior to any experimental efforts.

Instead of trying to estimate the abundance of all genomes, we cluster them into groups, and replace them by the group centroid genomes, as a representative of each group. The centroid is the one with the smallest sum of distances to all the others in the same group. This basically means we get fewer columns and rows in ***X***. We employed a clustering procedure as follows:

1. Compute the correlation distance between all pairs of genomes from the columns of ***X***.
2. Compute a single linkage hierarchical clustering of the genomes based on this. This results in a dendrogram.
3. Each height in the dendrogram corresponds to an alternative clustering. Choose the largest dendrogram height resulting in a copy number matrix with condition value below a user-specified tolerance.

In this way, the user specifies a tolerated upper condition value (e.g., 100 or 1000), and genomes will be clustered to the finest resolution not violating this. A larger tolerance value leads to a finer resolution, but also more unstable estimates.

### Simulation study

We also included a simulation study, where we compared the RMS approach to shotgun metagenome sequencing at various resolutions. Genome similarity was computed as whole-genome p-distance (i.e., 1.0 minus the Average Nucleotide Identity (ANI)). A whole-genome p-distance of 0.0 means identical genomes and above 0.3 means very different genomes. Strains from the same species usually have p-distance below 0.05. In most real communities, like the human gut, we must expect some closely related strains, having a p-distance of well below 0.05.

In [32], 1520 genomes from the human gut were isolated and sequenced. The whole-genome p-distances between all pairs of these genomes were computed using the MASH software [19], and then used to form clusters at three different resolutions: p-distances 0.05, 0.02, and 0.01. The cluster centroids were used as community members. The following procedure was applied to all communities, separately: from a community of *G* genomes, a sample contained reads from 100 randomly selected genomes (i.e., 100 of the *G* genomes are present, the remaining *G-*100 are absent). Their abundances were exponentially distributed such that the largest abundance was 100 times the lowest abundance (dynamic range of 100), see supplementary figure 1. Let *f*_*1*_*, f*_*2*_*,...,f*_*G*_ be the relative abundance for each of the *G* genomes in the community (i.e., 100 of them are positive and the rest are zero, and they all sum to 1.0). These values form the actual relative abundances that we later tried to estimate.

This was repeated 25 times for each community, forming 25 different samples. Note that for each sample, new 100 present genomes were randomly selected from the sub-population, thus different genomes were present/absent in each sample.

Reads were simulated using the ART software [39], using Illumina HiSeq 2500 error profiles, resulting in paired-end reads of 150 bases. For each sample, we simulated 1 million read pairs, either as a shotgun sample or as an RMS amplicon sample.

### Shotgun data

The ART software requires the user to supply the reference sequences to simulate from as well as the number of read pairs to generate. In shotgun metagenome sequencing, the probability of a read pair to originate from genome *g* is proportional to the abundance of the genome multiplied by its size. After fragmentation of the genomic DNA, the reads are sampled from this fragment pool, and larger and more abundant genomes will contribute with more fragments. Thus, if *z*_*g*_ is the size of genome *g*, we form a weight for genome as

$$ {\mathrm{w}}_{\mathrm{g}}={\mathrm{f}}_{\mathrm{g}}\ {\mathrm{z}}_{\mathrm{g}} $$

Given that we sequenced a million read pairs, these were spread out among the genomes by random sampling using the probabilities

$$ {p}_g={w}_g/{\sum}_{j=1}^G{w}_j $$

resulting in read counts *r*_*1*_*, r*_*2*_*, ...,r*_*G*_ for each genome. Note that genomes with zero abundance get zero reads. Finally, read pairs were simulated from each genome, given these read counts, and assembled into a pair of fastq files. This was then repeated for each sample, producing new sets of fastq files.

### RMS data

Instead of random fragmentation of the genomic DNA, the RMS protocol results in amplicons based on the fragments we get from restriction enzyme cutting. For each genome sequence, we collected the RMS fragments *in silico*, again using the EcoRI and MseI restriction enzyme cutting motifs. Next, we have observed two main biases in how the RMS fragments from a given genome contributes to the pool of sequenced amplicons:

First, there is a length bias, especially very long fragments are poorly amplified. Let *l*_*gk*_ be the factor that scales the amplification of fragment *k* in genome *g*. This is a function of fragment length only, and in supplementary figure 2, we show the function we used for simulating this.

Second, we have also observed that some fragments are consistently more or less represented in the reads from a given genome. We denote this the fragment bias. Let *v*_*gk*_ be this fragment bias factor for fragment *k* in genome *g* (i.e., it may scale the amplification of fragment *k* up (*v*_*gk*_ *>* 1) or down (*v*_*gk*_ *<* 1)). These factors were sampled at random from the distribution in supplementary figure 2, once for each genome, and then used forever after. Both this distribution as well as the length bias function were estimated from real RMS data, using the restriction enzymes described above.

Together, this means that the fragments from genome g get the weights

$$ {\mathrm{w}}_{\mathrm{k}}={\mathrm{f}}_{\mathrm{g}}\ {\mathrm{l}}_{\mathrm{g}\mathrm{k}}\ {\mathrm{v}}_{\mathrm{g}\mathrm{k}} $$

where *k = 1,2,...F*_*g*_*,* and *F*_*g*_ is the number of fragments in genome *g*. All fragments, together with their weights, were assembled for all abundant genomes, and the read count for each fragment/amplicon was sampled at random, again using probabilities *p*_*g*_ *= w*_*g*_ */*${\sum}_{j=1}^G{w}_j$.

Note that for shotgun data, the weights are only affected by genome abundance and size, while RMS data is affected by genome abundance, number of fragments, length distribution, and fragment bias distribution for the present genomes.

### Databases

The databases contained all *G* genomes of the community, both the 100 present at various levels and the *G*-100 absent. For each community, all RMS fragments were found in all *G* genomes, and a copy number matrix was constructed using a 0.99 identity threshold, as described above.

For the shotgun data, we used the Kraken2 software [17] to obtain relative abundance estimates. This tool has shown good results in several benchmarking studies [40–42], but more importantly, is equipped with excellent facilities for building a custom database. In order to make a fair comparison to the RMS approach, the database of reference genomes must be the same as in the RMS case. Thus, custom Kraken2 databases were constructed, containing all *G* genomes of the respective communities. Also, the taxonomy was extended correspondingly, to have a taxonomy ID for every single genome, making it possible for Kraken2 to list hits to each genome.

### Analysis

The analysis of the RMS data was carried out as described above, but without any genome clustering, resulting in an estimate of the relative abundance of every genome in the database.

For the shotgun data, Kraken2 and its custom database was used to assign reads to the genomes, using the default confidence level of 0.0. Only reads assigned to the genome level were counted, since this is our focus. The read count for a genome was divided by the genome size (base pairs), to produce the genome signal. Finally, these signals were divided by the total sum of signals, to produce relative abundances for all genomes in the communities.

## Supplementary Information

**Additional file 1: Supplementary figure 1.** All simulated samples contained reads from 100 randomly selected genomes, and their relative abundances in the sample were according to this barplot. The largest abundance is 100 times the smallest. Different genomes were selected as the most/least abundant and absent ones in each sample, but this abundance distribution was used every time.

**Additional file 2: Supplementary figure 2.** In order to simulate RMS data, some known biases were introduced to the signals. The upper panel shows the fragment-length bias used. All signals were scaled by this function, i.e. fragments of length around 200 bases remained close to unchanged (scale $1.0$) while signals from shorter or longer fragments were scaled down. The lower panel shows the fragment-bias distribution. For each fragment within a genome, a factor was sampled from this distribution, and the signals from the fragments were scaled accordingly. The mean value of this distribution is $1.0$, but some fragments may have signals up to six times as large, or down to almost nothing. Both the length-bias function and the fragment-bias distribution were estimated from real RMS data.

## Data Availability

The mock datasets generated and analyzed during the current study are available in the Sequence Read Archive repository, under the accession PRJNA574678; see https://www.ncbi.nlm.nih.gov/bioproject/PRJNA574678/. The computational methods described in this paper are available as an R-package. It is currently available at GitHub, together with some tutorials describing the analysis steps; see https://github.com/larssnip/microRMS.
