# Supplementary figures and images for: Reduced metagenome sequencing for strain-resolution taxonomic profiles
Source: Microbiome. 2021 Mar 29;9:79. doi: 10.1186/s40168-021-01019-8 (PMC8008692; doi:10.1186/s40168-021-01019-8)

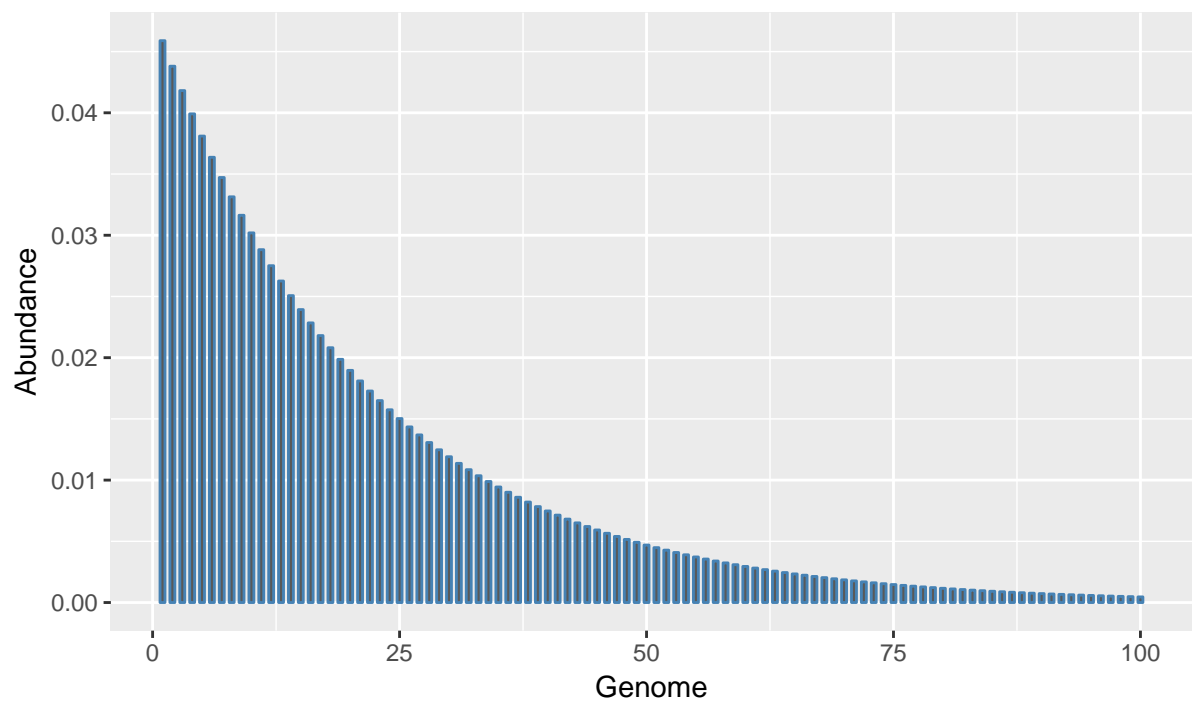

Supplement: Supplementary file 2 — Additional file 1: Supplementary figure 1. All simulated samples contained reads from 100 randomly selected genomes, and their relative abundances in the sample were according to this barplot. The largest abundance is 100 times the smallest. Different genomes were selected as the most/least abundant and absent ones in each sample, but this abundance distribution was used every time. [file 40168_2021_1019_MOESM2_ESM.pdf]

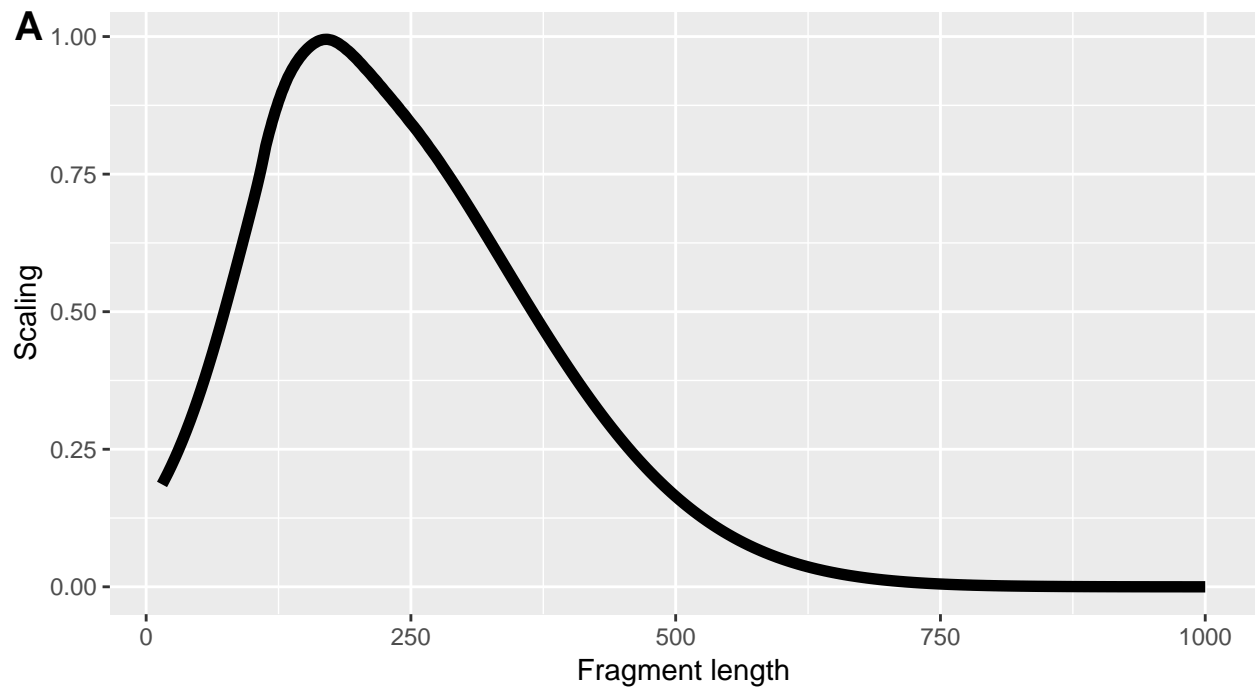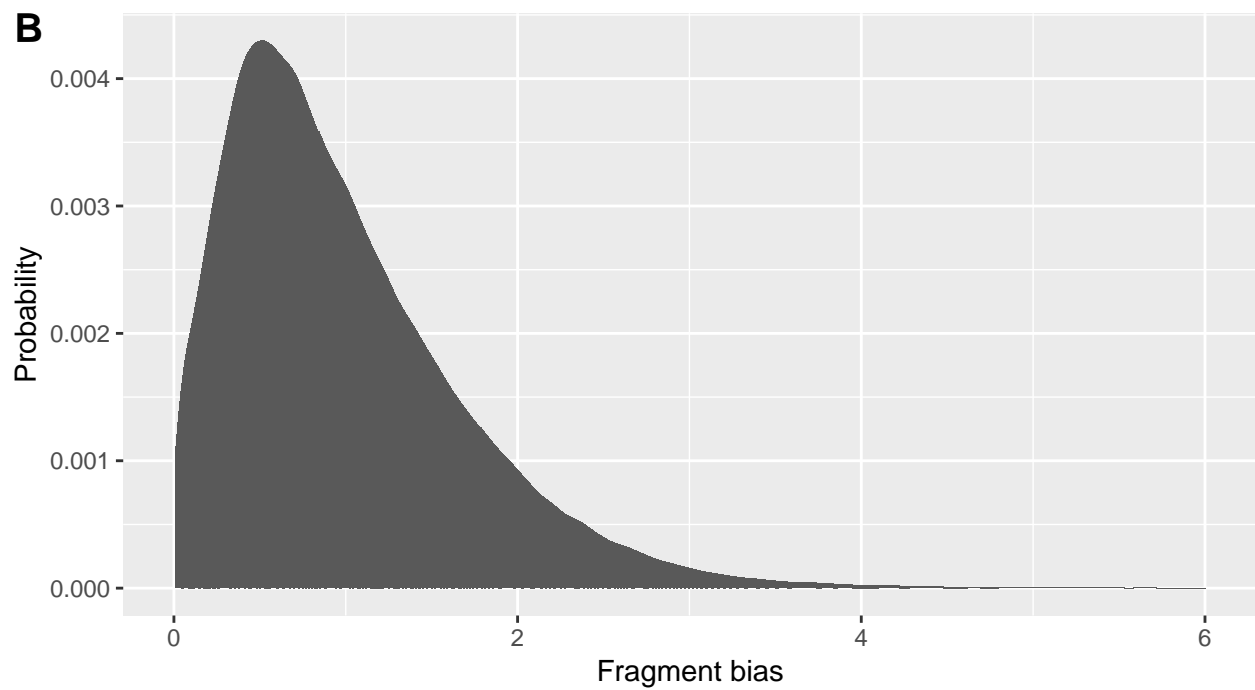

Supplement: Supplementary file 3 — Additional file 2: Supplementary figure 2. In order to simulate RMS data, some known biases were introduced to the signals. The upper panel shows the fragment-length bias used. All signals were scaled by this function, i.e. fragments of length around 200 bases remained close to unchanged (scale $1.0$) while signals from shorter or longer fragments were scaled down. The lower panel shows the fragment-bias distribution. For each fragment within a genome, a factor was sampled from this distribution, and the signals from the fragments were scaled accordingly. The mean value of this distribution is $1.0$, but some fragments may have signals up to six times as large, or down to almost nothing. Both the length-bias function and the fragment-bias distribution were estimated from real RMS data. [file 40168_2021_1019_MOESM3_ESM.pdf]
